# Oral Microbes and Mucosal Dendritic Cells, “Spark and Flame” of Local and Distant Inflammatory Diseases

**DOI:** 10.3390/ijms21051643

**Published:** 2020-02-28

**Authors:** Mohamed M. Meghil, Christopher W. Cutler

**Affiliations:** Department of Periodontics, The Dental College of Georgia at Augusta University, Augusta, GA 30912, USA; mmeghil@augusta.edu

**Keywords:** Dendritic cells, immunology, periodontitis

## Abstract

Mucosal health and disease is mediated by a complex interplay between the microbiota (“spark”) and the inflammatory response (“flame”). Pathobionts, a specific class of microbes, exemplified by the oral microbe *Porphyromonas gingivalis*, live mostly “under the radar” in their human hosts, in a cooperative relationship with the indigenous microbiota. Dendritic cells (DCs), mucosal immune sentinels, often remain undisturbed by such microbes and do not alert adaptive immunity to danger. At a certain tipping point of inflammation, an “awakening” of pathobionts occurs, wherein their active growth and virulence are stimulated, leading to a dysbiosis. Pathobiont becomes pathogen, and commensal becomes accessory pathogen. The local inflammatory outcome is the Th17-mediated degenerative bone disease, periodontitis (PD). In systemic circulation of PD subjects, inflammatory DCs expand, carrying an oral microbiome and promoting Treg and Th17 responses. At distant peripheral sites, comorbid diseases including atherosclerosis, Alzheimer’s disease, macular degeneration, chronic kidney disease, and others are reportedly induced. This review will review the immunobiology of DCs, examine the complex interplay of microbes and DCs in the pathogenesis of PD and its comorbid inflammatory diseases, and discuss the role of apoptosis and autophagy in this regard. Overall, the pathophysiological mechanisms of DC-mediated chronic inflammation and tissue destruction will be summarized.

## 1. Introduction

### 1.1. Dendritic Cells

Dendritic cells (DCs) are professional antigen-capture and -presenting cells (APCs) that play an important role in the innate immune system and serve as a bridge to the adaptive immune response [1]. Among total peripheral blood mononuclear cells (PBMCs), DCs generally constitute a very low frequency in individuals, around 1%. DCs are classically recognized as antigen-presenting leukocytes, lacking other leukocyte lineage markers (CD3, 14, 15, 19, 20, 56) with expression of high levels of major histocompatibility complex (MHC) class II (HLA-DR) molecules (lineage−HLA-DR+) [2]. Human DCs are generally divided into two major subpopulations, the plasmacytoid (pDC) (CD11c^−^CD123^+^) and myeloid (mDC) (CD11c^+^CD123^−^) lineages. mDCs can be further subdivided into CD141 (BDCA-3)^+^, CD16^+^ DC and CD1c (BDCA-1)^+^ DC subsets [3,4,5]. Human DCs originate from CD34^+^ haematopoietic precursors in the bone marrow that give rise to monocyte-DC progenitors with the ability to further develop into monocytes, or the common DC progenitor [6]. The common DC progenitor develops into pDC and pre-cDC that migrate via blood and peripheral lymphoid tissues. The pre-cDC differentiate into CD1c^+^ and CD141^+^ DC subsets [7]. pDCs play an important role in responding to viral infection by producing large amounts of type-I interferon. Due to the expression of the HIV receptors CD4, CCR5, and CXCR4 on pDCs, these cells can efficiently transfer the virus [8]. However, mDCs are reported to be important in both the clearance and processing of bacterial pathogens and apoptotic cells, along with the associated antigen presentation [5]. 

Immature DCs are equipped with a plethora of pattern-recognition receptors (PRRs), present on the cell surface and intracellularly, that capture a wide variety of microbes in the peripheral tissues, including the periodontium. PRRs are designed to identify pathogens via innate recognition of pathogen-associated molecular patterns (PAMPs), such as lipopolysaccharide (LPS), fimbriae, or flagellin [9]. Upon pathogen recognition, PRRs signal to the host the presence of infection and trigger antimicrobial and proinflammatory responses by activating a multitude of intracellular signaling networks, including adaptor molecules, kinases, and transcription factors. PRR-activated signal transduction pathways ultimately result in the activation of gene expression and production of a broad range of molecules, including cytokines, chemokines, cell adhesion molecules, and immunoreceptors, which together not only orchestrate the early host response to invading pathogens, but also represent an important link to the adaptive immune response [9]. This natural mechanism of the innate immune response is designed to enhance the ability of the host immune response to prevent infection, eliminate invading pathogens and stimulate the acquired immune response. PRRs can be classified into five superfamilies, based on protein domain homology: Toll-like receptors (TLRs), C-type lectin receptors (CLRs), nucleotide-binding domain, leucine-rich repeat (LRR)-containing (or NOD-like) receptors (NLRs), RIG-I-like receptors (RLRs), and the AIM2-like receptors (ALRs). These families can be further broken down into two main categories: membrane-bound receptors and unbound intracellular receptors. The former category consists of the TLRs and CLRs, which are found at the cell surface or on endocytic compartments. These receptors recognize microbial ligands in the extracellular environment and within endosomes. On the contrary, NLRs, RLRs, and ALRs are located in the cytoplasm, where they survey for the presence of intracellular pathogens [10]. DCs are endowed with a particularly diverse set of PRRs that enable them to recognize a wide variety of microbes in the periphery. Among the CLR, is Dendritic Cell-Specific Intercellular adhesion molecule-3-Grabbing Non-integrin (DC-SIGN). 

DC-SIGN, also known as CD209, is a cell surface receptor that belongs to the CLR family. Early reports identified DC-SIGN from human placenta as a receptor for the HIV-1 envelope glycoprotein gp120 [11]. Subsequently, DC-SIGN was identified as a cell-adhesion molecule whose expression was thought to be restricted to DCs and enhances infection of CD4+ T cells by HIV [12]. Multiple in vitro studies of DC-SIGN indicate it has multifunctional properties, including in intracellular communication, cell migration, pathogen recognition and capture, cell signaling, and antigen presentation. DC-SIGN is a type II membrane receptor that contains a C-terminal carbohydrate-recognizing domain. Early studies showed that the DC-SIGN C-terminal carbohydrate recognizing domain preferentially binds to the high-mannose N-linked oligosaccharides GlcNAc (N-acetylglucosamine) and Manα1-3[Manα1-6] Man (mannose), but later it was shown that DC-SIGN also binds to fucose-containing Lewis blood group antigens, which are also heavily expressed by microorganisms [13,14]. The results by Applemelk et at. have identified DC-SIGN as a novel pathogen receptor on human DC for *Mycobacterium tuberculosis (M. tuberculosis)*, *Helicobacter pylori (H. pylori)*, *Leishmania mexicana*, and *Schistosoma mansoni*, potentially implicated in inducing pathogen-mediated inflammatory response [13]. Several studies by Zeituni et al. of the oral pathogen *Porphyromonas gingivalis* (*P. gingivalis*) reported that its native minor fimbria is glycosylated with DC-SIGN targeting sugars (fucose, mannose, galactose, and N-acetylglucosamine) that enable it to bind to DC-SIGN on DCs [15]. In addition to pathogen recognition, DC-SIGN is also involved in cellular interactions between DCs and other immune cells. For example, interactions between DC-SIGN and both CEA-related cell adhesion molecule 1 and Mac 1 is required for cellular interaction between DCs and neutrophils, promoting T cell proliferation and inducing Th1 cell response [16]. Moreover, DC-SIGN promotes IL-2 production by CD3-activated T cells [17] and enhances DC-facilitated B cell response against microbial DNA [18]. 

DC-SIGN plays a crucial role in innate immunity. Certain pathogens escape immune surveillance and survive inside DCs. Such mechanism is attributed to dampening of the DCs response by DC-SIGN. Several studies have shown that *P. gingivalis*, Ebola virus, Hepatitis C virus, Cytomegalovirus, HIV-1, *M. tuberculosis*, *H. pylori*, *Streptococcus pneumoniae*, and many parasites and tumors all interact with DC-SIGN [15,19,20,21,22,23,24,25,26,27]. HIV-1 gp120 binds to DC-SIGN and facilitates HIV-1 capture by DCs at entry sites, transporting it to lymphoid tissues in which HIV-1 is transmitted from DCs to CD4+ T cells [22]. In addition, HIV-1 reportedly hijacks the process of initiating the adaptive immune response, transmitting the virus from the mucosa to the secondary lymphoid organs [28]. Moreover, DCs infected with HIV-1 show resistance to NK-induced TRAIL-mediated apoptosis [29]. *M. tuberculosis* also can manipulate the DC-mediated immune response and escape immune surveillance. 

### 1.2. DCs of the Oral Mucosal Tissues

**Figure 1:**
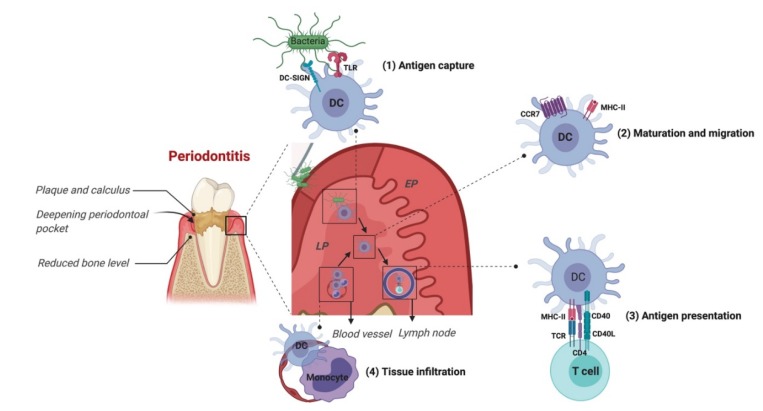
Microbial-dendritic cell (DC) interactions in diseased tissues of periodontitis. DCs recognize and uptake dysbiotic invasive pathogen *Porphyromonas gingivalis* by a combination of TLR2 and DC-SIGN. DCs migrate through lamina propria (LP) and undergo maturation process and acquire migratory profile. Semi-mature DCs ‘stall’ in the tissues, while fully mature DCs migrate to secondary lymphoid organs (SLO) to present antigens in the context of MHC molecules to T cells and activate the adaptive immune response. As maturing DCs efflux from tissues, blood inflammatory DCs and monocytes migrate into the tissues and differentiate into DCs to replace migrating DCs and maintain proper DCs homeostasis.

DCs infiltrate several mucosal sites, including oral mucosa. Previous studies have shown that DCs actively mobilize in and out of oral mucosal tissues at different stages of periodontal health and disease [30,31,32,33], particularly increasing in the lamina propria of periodontitis tissues. DCs infiltrate the peripheral tissues in the immature state, where they capture foreign bodies and pathogens, but also debris and apoptotic cells. Upon pathogen recognition and capture, immature DCs undergo maturation and must process the antigens and present them on surface MHC-II molecules to activate CD4+ T cells. In addition, mature DCs are capable of activating CD8+ T cells by presenting antigens on MHC-I molecules. Due to their ability to present antigens via both MHC class I and II, mature DCs are described as professional antigen presenting cells (APCs). The process of DC maturation involves up-regulation of costimulatory molecules (CD80, CD86), maturation markers (CD83), and antigen presenting molecules (MHC class I and II). Mature DCs also acquire a high migratory profile via upregulation of chemokine receptors (e.g., CCR7) and secretion of cytokines (e.g., IL-12p70) [34]. Under optimum conditions, DCs migrate to secondary lymphoid organs and present the captured antigens to T-lymphocytes. As DCs migrate to lymphoid organs, blood DCs and monocytes migrate into the tissues and differentiate into DCs to replace migrating DCs and maintain proper DCs homeostasis (Figure 1). 

**Figure 2:**
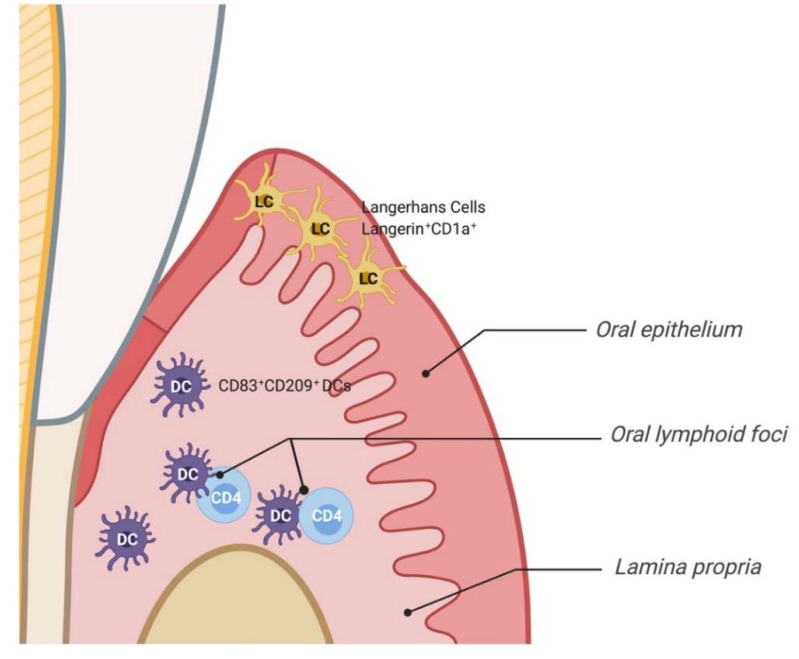
DC subsets in human periodontal tissue. Langerhans cells (LC) infiltrate the oral epithelium (EP). The lamina propria (LP) is infiltrated by mature DCs, where they form oral lymphoid foci (OLF) with CD4^+^ T cells resulting in T cell activation and cytokines secretion.

DC subsets are recognized by anatomical location, function, and expression of specific markers. The distinct microenvironment in the oral mucosa contributes to the diverse distribution of DCs at different sites in the oral cavity. DC subset of the oral epithelium are referred to as Langerhans cells (LC), which express the C-type lectin langerin/CD207. LCs express different markers in humans than in mice. In humans, immature LCs expressing langerin and CD1a predominantly infiltrate the gingival epithelium, whereas in mice they are identified by expression of langerin, CD11c, MHC class II and Ep-CAM. Other DC subsets reside in the lamina propria. In mice—based on the expression of CD11c, CD11b, MHC class II, CD103, and langerin—lamina propria DCs are divided into 4 subsets: (i) (CD11c^+^-CD11b^+^- MHC class II^+^) interstitial DCs, (ii) (CD11c^+^-CD11b^+^- MHC class II^+^-CD103^+^), (iii) (CD11c^+^-CD11b^+^- MHC class II^+^-CD103^+^-langerin^+^) DCs, (iv) (CD11c^−^-CD11b^+^- MHC class II^+^). The latter subpopulation is referred to as macrophage-like cells because of their expression of F4/80. However, in humans, CD83^+^ mature DCs are localized in the lamina propria of healthy and PD tissues, infiltrating the CD4+ lymphoid-rich lamina propria, forming oral lymphoid foci in situ [33] (Figure 2, Table 1). 

### 1.3. Periodontitis and Dendritic Cells

Periodontitis (PD) is a chronic, dysbiotic inflammatory disease that affects almost half of the population in the United States. The disease leads to destruction of the tooth-supporting tissues, also known as the periodontium, and consists of gingiva, periodontal ligament, and alveolar bone. PD elicits a nonprotective immune response, with an early massive influx of polymorphonuclear leukocytes (PMNs) in response to the dental plaque or biofilm. Tissue destruction is attributed to the increased influx, activation, and lysis of PMNs attracted to the site of the disease. PD is preceded by a condition termed gingivitis, which is characterized by inflammation confined to the gingival tissue without destruction of the underlying soft and hard tissue. Despite the histopathological similarities between gingivitis and PD, there is no clear evidence that explains the mechanism by which gingivitis lesions proceed to PD lesions. The periodontal environment provides opportunity for commensal and pathogenic oral microbes with a rich niche to live in. In healthy conditions, the microbial composition of dental plaque biofilm is balanced and stable. When this balance is disrupted by inflammation, microbial homeostasis breaks down and periodontal disease occurs. The progression from gingivitis to PD is associated with a dramatic shift from a symbiotic microbial community structure, comprised mainly of aerobic bacteria; to a dysbiotic, anaerobic microbial biofilm [35]. Anaerobic bacterial complex consisting of *P. gingivalis*, *Treponema denticola* (*T. denticola*), and *Tannerella forsythia* (*T. forsythia*), also known as the red complex, has been implicated in the development of PD [36]. Although PD is an infection-initiated inflammatory disease, the presence of these microbes alone (the “spark”) is not sufficient to develop the disease. It has been established that periodontal tissues destruction is driven by the host immune response (the “flame”) via production of proinflammatory cytokines [35]. The disease starts by the accumulation and adhesion of the bacterial plaque on the tooth surface. The innate immune system takes over the fight against the bacterial plaque as a first line of defense through its components: anatomical and physical barriers, secretory molecules, and cellular elements such as neutrophils, macrophages, and DCs. LCs may play a regulatory role in experimental periodontitis. In an experimental model of periodontitis in mice, mucosal LCs induced differentiation of *P. gingivalis*-specific Th17 [37]. 

**Figure 3:**
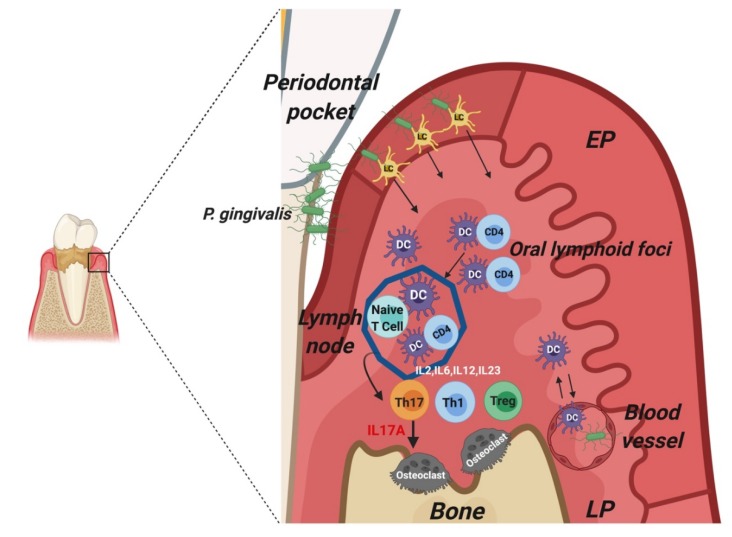
Role of mature DC-CD4+ T cells clusters in osteoclastogenic response. Mature CD83+ MHCII+ DCs in lamina propria (LP) engage with CD4+ T cells and elicit destructive recall responses. Mature DCs and other antigen presenting cells (APCs) release IL-6, TNFα, IL-1β, and IL-23, promoting differentiation of T cells into IL-17A+ Th17 cells. Th17 cells express RANK-L, release IL-17A, IL-17F, and IFN which promote differentiation of preosteoclasts into Trap+ osteoclasts.

Initiation and regulation of the adaptive immune response is principally mediated by DCs, in the proper cytokine microenvironment. DCs play an important role in instructing naïve T cells to differentiate into Th1, Th2, Th17, follicular helper T (Tfh), and T regulatory cells (Treg). Th1 cells are mainly involved in cellular immunity and produce interleukin (IL)-2 and interferon gamma (IFN-γ) [38]. Th2 cells produce IL-4, IL-5, and IL-13, and are mainly responsible for production of pathogen-specific antibodies via the activation of B cells [39]. The role of Th17 cells in periodontal environment is to increase neutrophil recruitment to the dental plaque for efficient bacterial clearance [40]. On the other hand, Treg cells down-regulate T-cell response and reduce excessive proinflammation and alveolar bone loss [41]. In periodontitis, DCs activation of T-cells takes place at the secondary lymphoid organs as well as in the lamina propria at the oral lymphoid foci (Figure 3). Baker et al. reported that in the absence of MHC class-II-responsive CD4+ T cells, *P. gingivalis* fails to induce periodontitis and alveolar bone loss. In addition, the lack of proinflammatory cytokines such as IFN-γ and IL-6 decreases alveolar bone loss in mice [42].

Recent studies suggest that dissemination of microbes from the periphery, through the systemic circulation to distant sites, could be occurring inside highly migratory DCs [43]. One of the proposed mechanisms is invasion of DCs, followed by manipulation of intracellular signaling in DCs. It has been shown that targeting of DC-SIGN on DCs by *P. gingivalis* minor fimbria facilitates invasion and results in extended survival of host DCs, through inhibition of apoptosis. P. gingivalis survives in DCs through evasion of autophagy. Fimbriae are appendages that are involved in *P. gingivalis* cell membrane and greatly contribute to its virulence [44]. *P. gingivalis* fimbriae play a crucial role in nearly all interactions between the bacterium and the host, as well as with other bacteria. More importantly, *P. gingivalis* fimbriae have been identified as a key factor in its adhesion, invasion, and colonization of the oral mucosa [15,45]. *P. gingivalis* is generally considered to have two distinct types of fimbriae; long and short fimbriae [46,47]. The long fimbriae (FimA) is also known as major fimbriae, while the short fimbriae (mfa-1) is known as minor fimbriae. Both *P. gingivalis* fimbriae are involved in initial attachment and organization of biofilm and attachment to other bacteria [48]. Most interestingly, the fimbriae of *P. gingivalis* have been shown to be important for invasion of DCs, particularly the minor fimbriae, comprised of a 67-kDa glycoprotein that is encoded by the *mfa1* gene [49]. Mfa1 targets the C-type lectin DC-SIGN on DCs for entry [47] and survival within [43]. The major fimbriae are composed of a 41-kDa protein called fimbrillin, encoded by the *fimA* gene [50]. It has also been reported that CD1c^+^(BDCA-1) CD209^+^ blood myeloid DCs expand in subjects with PD, relative to healthy controls. In addition, this expansion further increases 24 h after mechanical debridement (scaling and root planning), attributed to bacteremia [43]. Furthermore, myeloid DCs have been shown to be increased in PD patients with existing coronary artery disease. Not only do DC populations increase in systemic circulation of PD subject with coronary artery disease, but it was also revealed that these DCs carry microbial cargo, including *P. gingivalis* and other species. Using immunohistochemical studies, postmortem analysis of coronary artery samples shows colocalization of myeloid DCs marker, CD209 (DC-SIGN), with *P. gingivalis* minor fimbria protein (mfa-1) in the atherosclerotic plaques of coronary-artery-diseased patients. Epidemiologic studies have linked the association of PD with cardiovascular diseases, but the mechanism remains unclear. 

### 1.4. Apoptosis

Apoptosis, or programmed cell death, is essential in eliminating unwanted cells and cell turnover, but can be a target for pathogens in order to preserve their host cell [51]. There are two major apoptosis pathways, the intrinsic and the extrinsic pathway. The intrinsic pathway is stimulated by intracellular signals and governed by B-cell lymphoma 2 (BCL-2) family of proteins, wherein the mitochondria plays an important role. The mitochondria is stimulated by the proapoptotic members of BCL-2 family to release molecules that can influence apoptosis [52]. Upon its release from the mitochondria, cytochrome *c* promotes formation of the apoptosome, which comprises apoptotic protease-activating factor 1 (APAF1), pro-caspase 9, and cytochrome *c*. Caspase 9 is activated by the apoptosome then cleaves pro-caspase 3 to form active caspase 3 [53]. Alternatively, the extrinsic pathway is triggered by external signals and stimulated when a death-inducing factor such as FAS ligand (FASL) binds to its receptor (FAS) and recruits the adaptor FAS-associated death domain protein (FADD) and pro-caspase 8, forming the multiprotein, death-inducing signaling complex (DISC). Pro-caspase 8 is processed into mature activated caspase 8 in the DISC. The activated caspase 8 then processes pro-caspase 3 to form active caspase 3 [54,55,56]. Both the extrinsic and intrinsic pathways lead to caspase 3 activation, which subsequently cleaves more than 500 cellular substrates to execute the apoptosis process. 

### 1.5. Defective Apoptosis and Risk of Autoimmunity and Microbial Dissemination

Although they have a similar lifespan upon maturation, circulating DCs are short lived when compared to tissue resident DCs [57]. Abnormal DC survival and defective apoptosis can lead to their accumulation, persistent lymphocyte activation, and autoimmunity, which is consistent with the critical role of these cells in maintaining immune self-tolerance [58]. DC apoptosis can be disrupted by activation of signaling through surface receptor ligands or by infection by certain pathogens [59]. Control of immune cells’ lifespan and turnover is an important facet of immune homeostasis and tolerance. Interestingly, mutations in *lpr* (lymphoproliferation) and *gld* (generalized lymphoproliferative disease) have been shown to result in enlargement of lymph nodes and spleen. In mice, these loss-of-function mutations lead to increased development of systemic lupus erythematosus (SLE). It is noteworthy that the *lpr* and *gld* phenotypes are the result of loss-of-function mutations in Fas (Fas^lpr^) and Fasl (Fasl^gld^), respectively [60,61]. Furthermore, patients with autoimmune lymphoproliferative syndrome (ALPS) have been shown to carry somatic or germline mutations in the genes that encode FAS, Fas-ligand, caspase 10, caspase 8, NRAS, and KRAS [62,63,64]. Defective apoptosis not only affects cellular immunity, but also the humoral arm of the immune system. The *lpr* and *gld* mice were shown to produce self-reactive antibodies, and it was found that the FAS system has a regulatory role in eliminating autoreactive B cells [65]. Bim is a proapoptotic member of the BCL2 family of proteins that is involved in regulating the intrinsic pathway of apoptosis. It has been reported that Bim-deficient mice develop an SLE-type autoimmune disease and show lymphocytes that are refractory to different apoptosis stimuli [66]. In addition, Bim plays an important role in regulating the lifespan of short-lived immune cells such as eosinophils, neutrophils, and monocytes [67]. Targeting DC-SIGN receptor in DCs by *P. gingivalis* has also been shown to inhibit apoptosis in DCs via manipulating the Akt-FOXO1 pathway, which is an important regulator of the apoptosis process. Upon phosphorylation, Akt translocates to the nucleus where it phosphorylates FOXO1. As a result, phosphorylated FOXO1 (p-FOXO1) translocates from the nucleus to the cytoplasm, where it undergoes proteosomal degradation, leading to inhibition of apoptosis, as FOXO1 is an important transcription factor involved in the regulation of the expression levels of proapoptotic genes such as Bim. DC infection by *P. gingivalis* significantly increases level of expression of phosphorylated Akt (p-Akt), increases nuclear translocation of p-Akt and cytoplasmic translocation of p-FOXO1, and ultimately decreases the levels of expression of proapoptotic proteins and extends the survival of DCs [68]. Not only does *P. gingivalis* disrupt homeostatic apoptosis in DCs [51], but it also disrupts DCs homing to secondary lymphoid organs via reprograming chemokine receptors on DCs, leading to transferring the inflammation to vascular sites [34]. Furthermore, disruption of FOXO1 function in DCs has been implicated in induction of alveolar bone loss and increases susceptibility to periodontitis. A study by Xiao et al. reported that mice that had FOXO1 gene deletion in DCs and that were infected with *P. gingivalis* showed more alveolar bone loss and more loss of connective tissue attachment compared to control mice [69]. Together, these studies suggest that the intrinsic and extrinsic apoptotic pathways play important, collaborative roles in maintaining immune homeostasis. 

### 1.6. Autophagy

Autophagy is another important intracellular pathway involved in mucosal homeostasis, which human pathogens can exploit for survival [70]. Autophagy is initiated by the formation of the phagophore, a cup-shaped structure, via recruitment of autophagy-related proteins (ATGs) to a specific subcellular site called the phagophore assembly site (PAS) and nucleation of an isolation membrane. The phagophore continues to expand by a gradual elongation of the curved isolation membrane around a portion of the cytosol, resulting in sealing of the isolation membrane into a double-membraned vesicle called the autophagosome, trapping the engulfed cytosolic content as autophagic cargo. The autophagosome then fuses with the lysosome via fusion of its outer membrane with the lysosomal membrane to form an autolysosome. This fusion results in the release of the autophagic membrane into the lysosomal lumen, which is followed by degradation of the autophagic cargo by the autolysosomal hydrolytic environment [71,72]. Core ATG proteins essential for autophagosome formation and delivery of autophagic cargo to the lysosomes are grouped into five complexes [73], based on their functional and physical interactions: (1) the ULK1 (Unc-51-like kinase 1) complex—the serine/threonine protein kinase ULK1, RB1-inducible coiled-coil protein 1 (FIP200), ATG13, and ATG101; (2) ATG9—the transmembrane core ATG; (3) the class III PI3K (PI3KC3) complex—the catalytic subunit vacuolar protein sorting 34 that converts PI into PI-3-phosphate (PI3P), Beclin 1, and general vesicular transport factor p115, associated with ATG14 in PI3KC3 complex I (PI3KC3–C1) or UV radiation resistance-associated gene protein in complex II (PI3KC3–C2); (4) WIPI (WD repeat domain phosphoinositide-interacting) proteins and their partner ATG2; and (5) two ubiquitin (Ub)-like proteins and covalent conjugation targets: the Ub-like ATG12 conjugates with ATG5 (ATG12-ATG5) which further forms a complex with ATG16L (ATG12-ATG5–ATG16L), and Ub-like ATG8 family proteins (ATG8s), which include the light chain 3 (LC3) subfamily and the γ-aminobutyric acid receptor-associated protein (GABARAP) subfamily, which establish conjugates with membrane-resident phosphatidylethanolamine (PE). Nucleation of the phagophore takes place at the PAS on membrane domains, termed omegasomes, originate from the endoplasmic reticulum (ER). These membrane domains are PI3P-enriched and marked by the PI3P-binding protein zinc-finger FYVE domain-containing protein 1 [74]. Other sites are also implicated as PAS such as ER-mitochondria and ER-plasma membrane contact sites, the Golgi complex, plasma membrane, and endosomes [75]. Phagophore formation involves PAS formation, activation of the ULK1 complex and the PI3KC3–C1, and the recruitment of ATG9-containing vesicles, contributing to the membrane expansion [76]. In the first step of phagophore nucleation, ULK1 complex is recruited to the phagophore nucleation site, a process believed to be mediated by the C-terminal domain of ULK [77] and a specific amino acids cluster in the N-terminus of ATG13 [78]. Subsequently, PI3KC3 is recruited to phagophore nucleation sites, possibly via the role of ATG14L, to generate PtdIns3P [79]. The association of the PI3KC3 complex with the ER membrane is further stabilized by the interaction between Beclin 1 and Vacuole membrane protein 1 (VMP1) [80]. Finally, the ATG12–ATG5–ATG16L1 complex is recruited to the phagophore, where it plays an important role in the process of LC3 or GABARAP lipidation [81]. Ub-like ATG8 family proteins are prominently implicated in phagophore expansion. The cysteine protease ATG4 processes pro-ATG8s to expose a glycine residue that is important for the conjugation of ATG8s to PE [82]. Upon processing, ATG8s are activated by the E1-like enzyme ATG7 and subsequently conjugated to membrane-associated PE via the activity of ATG3, converting it from a freely diffuse, unlipidated form (LC3-I) into a membrane-bound, lipidated form (LC3-II). Previous studies have reported that conjugation of ATG8s to PE promotes phagophore expansion and sealing [83]. In addition, WIPI2 binds to ATG16L1 directly, consequently recruiting the ATG12~ATG5–ATG16L1 complex that enhances the ATG3-mediated conjugation of ATG8s. ATG3 requires stimulation by E3-like activity of the ATG12~ATG5 conjugate, established by activation of ATG12 by ATG7 and conjugation to ATG5 by E2-like ATG10.

### 1.7. Autophagosome Maturation

After phagophore expansion and sealing, the autophagosome subsequently undergoes maturation, a process involved with clearance of most of the ATGs from autophagosomal outer membrane. Furthermore, autophagosome maturation requires recruitment of two kinds of machineries, one that mediates lysosomal delivery and another that is responsible for fusion with the lysosome. Autophagosome fusion with the lysosome requires SNAREs syntaxin 17 and synaptosomal-associated protein 29 on the autophagosome and vesicle-associated membrane protein 8 (VAMP8) on the lysosome [84]. In addition, SNARE-mediated fusion of the autophagosome to the lysosome is supported via the homotypic fusion and protein sorting (HOPS) complex, which mediates autophagosomal membrane tethering. ATG8s play a crucial role in the maturation of the autophagosome. They link the autophagosome to kinesins responsible for lysosomal delivery [85] and recruit the HOPS complex to the autophagosome [86].

### 1.8. Autophagy Regulation

Autophagy is induced by starvation and deprivation of amino acids, leading to inhibition of the serine/threonine kinase mechanistic target of rapamycin (mTOR) [87,88]. mTOR coordinates cell growth and maintain metabolic homeostasis by integrating signals from a wide variety of stimuli including stress, oxygen, amino acids, and energy. mTOR forms two distinct protein complexes in mammals, mTORC1 and mTORC2, but only mTORC1 is sensitive to nutrients and directly regulates the process of autophagy [89]. Rag GTPase complexes, members of the Ras family of GTPases, are involved in mTORC1 activation by amino acids, via tethering mTORC1 to the lysosomes [90]. The Rag family consists of four members (Rag A, B, C, and D) that establish heterodimers. In the presence of amino acids, a Rag dimer composed of an A/B subunit with a C/D subunit is recruited to the lysosome through a complex called the Regulator complex, where it binds to mTORC1 [90,91]. 

Autophagy may also be triggered by declining cellular energy, such as a decrease in glucose level. The process of cellular metabolic homeostasis and energy levels is sensed by ATP:ADP:AMP ratio intracellularly through the regulatory kinases 5′ AMP-activated protein kinase (AMPK) and serine/threonine-protein kinase STK11 (LKB1) [92]. LKB1 activates autophagy and inhibits mTORC1 indirectly downstream of AMPK via activation of the TSC2 (tuberous sclerosis 2) complex and directly by Raptor phosphorylation [93]. 

Several other proteins have been reported to regulate autophagy, including Beclin 1, which inhibits autophagy when inactivated. Binding of the antiapoptotic protein BCL2 to Beclin 1 and phosphorylation of Beclin 1 by Akt and EGFR (epidermal growth factor receptor) inhibit autophagy [94,95]. On the other hand, Beclin 1 phosphorylation by ULK1, MAPKAPK (mitogen-activated protein kinase−activated protein kinase) 2 and 3, AMPK, and DAPK (death-associated protein kinase) has been reported to promote autophagy [96,97,98,99]. In high-nutrient conditions, mTORC1 binds to and phosphorylates ATG13 and ULK1 [100]. mTORC1 phosphorylates the site Ser 757 on ULK1. Phosphorylation of ULK1 at Ser 757 is important for mTORC1 to inhibit ULK1 and repress induction of autophagy [89]. On the other hand, under starvation conditions, mTORC1 is inhibited and its phosphorylation sites on ULK1 are dephosphorylated. Simultaneously, ULK1 dissociates from mTORC1 and undergoes autophosphorylation followed by phosphorylation of FIP200 and ATG13 [101]. 

### 1.9. Autophagy in Infection and Immunity

Several reports have demonstrated the important role of autophagy in a plethora of immune functions. Autophagy is not only implicated in clearance of intracellular pathogens [102,103,104], but also in secretion of inflammatory cytokines [105], antigen presentation [106,107], and development of lymphocytes [108]. In addition, autophagy is regulated by a wide variety of immunological signals in response to ligands for PRRs such as TLRs and NLRs or to cytokines and respond to PAMPs and DAMPs. Moreover, TLR ligand-coated particles induces stimulation of phagocytes and LC3-PE conjugation. This process is called LC3-associated phagocytosis [109]. Xenophagy is a selective autophagy process targeting pathogens. Antibacterial xenophagy plays an important role in inducing immune response in phagocytes such as DCs, macrophages, and neutrophils. Phagocytes recognize bacteria by a wide range of PRRs expressed on the cell surface. Following bacteria internalization via phagocytosis, intracellular signaling activation leads to induction of immune response. Concomitantly, this phagocytosis process is normally accompanied by killing of the bacteria mediated by lysosomes and reactive oxygen species (ROS), as well as through antimicrobial peptides [110]. The interception of autophagy and microbes occurs at different stages of invasion. During bacterial infection, the autophagy and microbe interaction begins early by detecting microbial PAMPs via PRRs [111], continues by uptake of bacteria by host cell, and ends by autophagic adaptors [112]. Autophagy in DCs has been implicated in induction as well as prevention of autoimmune diseases. Alissafin et al. reported that inhibition of the autophagic machinery in DCs results in suppression of autoimmune responses, and that autophagy-deficient mice showed reduced autoimmunity via failure of DCs to prime autoantigen-specific CD4+ T cells [113]. Another report exhibited that mice with autophagy-deficient DCs have increased survival via slowing down autoimmune disease progression through dampening IFNα levels [114]. 

### 1.10. Autophagy, a Tool for Resistance or Susceptibility to Infection

Autophagy process and autophagy proteins play a crucial role in host defense against microbial infection. Several reports using different models have demonstrated that defective autophagy pathway increases the host susceptibility to infection. Shelly et al. have shown that, using *Drosophila* model, depletion of Atg18, Atg7, and Atg12 increased viral replication and mortality even from infection by nonlethal viruses such as Vesicular Stomatits Virus [115]. Using the same model, another study has reported that impaired autophagy increased susceptibilily to *Listeria monocytogenes* (*L. monocytogenes)* [70]. Other studies using a murine model have shown that knockout of Atg5 in macrophages and neutrophils increases susceptibility to *L. monocytogenes* infection [116] and neuron-specific Atg5 knockout increases susceptibility to infection by central nervous system Sindbis virus [117]. Studies on vitamin D3 have unraveled a potential link between nutrition, innate immunity, and regulation of autophagy during mycobacterial infection. Vitamin D3 deficiency is associated with increased susceptibility to *M. tuberculosis* infection. Several studies have reported the role of vitamin D in the induction of autophagy via regulating a multitude of signaling pathways essential for autophagy [118,119,120]. It has been shown that vitamin D3-associated autophagy plays a crucial role in innate immunity, infection, and inflammatory diseases [121]. Interestingly, analysis of PBMCs and gingival samples from PD subjects has revealed a significant decrease in the expression levels of key proteins and genes involved in regulation of autophagy, relative to healthy controls. Furthermore, Vitamin D3 supplementation results in restoring autophagy function by increasing the expression levels of the same proteins [122]. The influence of *P. gingivalis* fimbria on autophagy in DCs has been reported in multiple in vitro studies. *P. gingivalis* evades intracellular killing in DCs and this immune escape tactic involves targeting DC-SIGN by *P. gingivalis* minor fimbria. Previous studies have shown that *P. gingivalis* evades intracellular killing in DCs and this immune escape tactic involves targeting DC-SIGN by *P. gingivalis* minor fimbria [102]. The same study reported that DC-SIGN-dependent uptake of *P. gingivalis* by DCs results in lower intracellular killing and higher intracellular content of *P. gingivalis*. In addition, blocking DC-SIGN by HIV glycoprotein 120 reduces *P. gingivalis* survival inside DCs, but the mechanism of this phenomenon was unclear [102]. A recent report revealed that inhibition of autophagy in DCs by *P. gingivalis* involves targeting the Akt-mTOR pathway, which is an important regulator of autophagy [68]. *P. gingivalis* infection increases expression of p-Akt Ser473, p-mTOR Ser2448, p-Raptor Ser792, and p-ULK1 Ser757, all of which are important elements in mTOR-dependent autophagy inhibition [68]. 

## 2. Conclusions

We conclude that dendritic cells play an important role in clearing infecting microbes from oral mucosa and in initiating the adaptive immune response in secondary lymphoid organs. However, DCs are poorly equipped to deal with dysbiotic pathogens such as *P. gingivalis*, that infect them, evade autophagy, and disrupt homeostatic mechanism of apoptosis, thus leading to chronic inflammatory diseases such as periodontitis. Future research should be directed towards the development of small molecule inhibitors that can interfere with manipulation of intracellular signaling pathways by *P. gingivalis*, and thus restore homeostatic autophagy and apoptosis.

## Figures and Tables

**Table 1:** DC subsets in periodontal tissues of mice and humans.

Mice	Oral Epithelium DCs	Langerin^+^ -CD11c^+^ -MHC class II^+^ -Ep-CAM^+^ Langerhans cells
	Lamina propria DCs	D11c^+^-CD11b^+^- MHC class II^+^ interstitial DCs
		CD11c^+^-CD11b^+^- MHC class II^+^-CD103^+^
		CD11c^+^-CD11b^+^- MHC class II^+^-CD103^+^-langerin^+^ CD11c^−^-CD11b^+^- MHC class II^+^
		CD11c^−^-CD11b^+^- MHC class II^+^
Humans	Oral epithelium DCs	Langerin^+^-CD1a^+^ Langerhans cells
	Lamina propria DCs	CD83^+^-CD209^+^

## Abbreviations

*P. gingivalis*	*Porphyromonas gingivalis*
DCs	Dendritic cells
PD	Periodontitis
APCs	Antigen-presenting cells
PBMCs	Peripheral blood mononuclear cells
MHC	Major histocompatibility complex
pDC	Plasmacytoid dendritic cells
PRR	Pattern recognition receptors
PAMP	Pathogen-associated molecular patterns
LPS	Lipopolysaccharide
TLRs	Toll-like receptors
CTLs	C-type lectin receptors
DC-SIGN	Dendritic Cell-Specific Intercellular adhesion molecule-3-Grabbing Non-integrin
LCs	Langerhans cells
OLF	Oral Lymphoid Foci
PMNs	Polymorphonuclear leukocytes
Treg	T regulatory cells
ATGs	Autophagy-related proteins
mTOR	Mechanistic target of rapamycin
